# Alcoholic Liquors in the Practice of Medicine

**Published:** 1887-08

**Authors:** Eugene Foster

**Affiliations:** Augusta, Georgia


THE ATLANTA MEDICAL AND SURGICAL JOURNAL.

VOL. IV. AUGUST, 1887. No. 6.

### Original Communications.

### ALCOHOLIC LIQUORS IN THE PRACTICE OF MEDICINE.

READ BEFORE THE MEDICAL ASSOCIATION OF GEORGIA AT ITS THIRTY-EIGHTH ANNUAL SESSION IN ATLANTA, APRIL 21, 1887, BY EUGENE FOSTER, M. D., AUGUSTA, GEORGIA.

(CONTINUED FROM PAGE 271.)

"Is there sufficient justification in any therapeutic advantage for the existing use of alcoholic liquors by medical men?"

Gentlemen, it does seem to me that there can be but one answer to this question, and that answer is, yes. We have shown you by general consensus of medical testimony that alcoholic liquors constitute one of our chief defences in a majority of the most dangerous diseases affecting the human race, and that our materia medica furnishes us with no reliable substitute therefor, and I ask you if this fact does not furnish us every justification for continuing to use this valuable remedy? If the argument that evil uses of medicines must banish them from rational and legitimate prescription in medicine prevails, then we must give up chloroform, ether, hydrate of chloral, cannabis indicus, bromides of potassium, sodium and ammonium, opium, morphine, laudanum, even the paregoric which we give to the helpless baby in convulsions. Why on earth should we be expected to banish alcoholic liquors from our materia medica? It seems incredible, but we are seriously told that "the medical profession, in maintaining the therapeutic value of alcoholic liquors, furnishes serious obstacles in the way of the great moral reform which is agitating the world in connection with the effort to suppress the liquor traffic." I have no sympathy with any such view as this. It is unfounded in reason and in fact. In this discussion we are considering the value of alcoholic liquors as therapeutic agents and wholly independent of the question of intemperance. We are considering a scientific question which must be decided upon facts relating to the scientific view thereof; the physical, mental and moral aspects of intemperance do not enter into this effort to ascertain the scientific status of the question. That persons to whom spirituous liquors have been prescribed for medicinal purposes may abuse the prescription, and thereby pervert the value of the remedy in their individual instance by falling into the habit of intemperance, is undeniable, but such facts can in nowise detract from the therapeutic value of alcoholic liquors properly used, nor can they furnish any rational or legitimate argument against the fact that the medical profession, while contending for the therapeutic value of alcoholic liquors, deplore the existence of intemperance as sincerely as any class or profession of men on earth.

No, no, gentlemen, our grand profession as a body is not blamable for the habit of intemperance. Where individuals fall into the habit of intemperance in consequence of prescription of alcoholics by medical men, they do so by perverting the beneficent gifts of science to their selfish and wanton purposes. Alcohol is not the only beneficent therapeutic agent intended for the relief of man which we daily see converted into instruments or agents of disease by abuse instead of use. Every objection which can be brought against the therapeutic value of alcoholics because misapplied by man can equally be lodged against ether, chloroform, cannabis indicus, hydrate of chloral, bromides of sodium, ammonium and potassium, opium, morphine, laudanum, paregoric, cocaine, etc., through a long list of the most valuable and indispensable remedies known to scientific medicine. Why, gentlemen, if the abuse of a remedy is sufficient justification to call for its disuse, then the habit and undeniable necessity of eating food must be numbered with the things that were, for gluttony is productive of obesity with consequent fatty degenerations of vital organs of the body, yet because eating food may degenerate into the vice of gluttony, I hope this fact may not be used as a potent argument against the physiological necessity of food to sustain the human body, yet the latter argument would be as rational as that used against the therapeutic use of alcoholics because intemperance follows their prescription. The essay under review quoted Dr. B. W. Richardson, the great temperance advocate, but failed to quote the following from the same paper by Dr. Richardson: "On one hand, I am quite sure that the professors of medicine have as full right to use alcohol as they have to use any other medicinal agent. No clamor from the public ought to interfere with them in this part of their work. For myself, I claim the perfect right, and I exercise it according to my best judgment with entire independence. I have prescribed alcohol for abstainers of the purest type, as I have chloroform, ether and other medicinal substances. Against such legitimate use abstainers should not object, unless they can show by their learning that their objection is founded on sound physiological data, bearing directly upon the facts of the case in which their objection is raised." So say I, gentlemen, and this principle is all that I contend for. Be it remembered, just here, that Dr. Richardson is President of the British Medical Temperance Association, an association formed for the definite purpose of aiding the cause of suppression of intemperance. Now, have these abstainers ever offered the proof which their President, Dr. Richardson, frankly and manfully says is incumbent upon them to show? In 1881 this "British Medical Temperance Association" published the following statement:

"Up to the present time we have not been able to collect any statistics of comparison between our own practice and that of other physicians and surgeons who prescribe alcoholic drinks largely, but many of us can compare the results of our present practice with those which attended our practice when we were accustomed to administer such drinks very generally, and we believe that the results we now witness are better than they were before."

Remember, gentlemen, that these abstainers do not wholly eschew alcoholic liquors in their practice. In the same publication of this Association, in 1881, I find the following statement: "All the members of the association are total abstainers from the use of alcoholic beverages; there are at the same time no laws of the Association which prevent members from prescribing alcoholic drinks for the sick under their professional care. As a rule, members rarely prescribe alcohol medicinally; some never prescribe it at all; others (as the President) only prescribe it, when they think it is required, in the form of pure ethylic alcohol in systematic doses." The only difference between the British Medical Temperance Association and all other doctors is that its members prescribe alcoholic liquors for the sick under their professional care when they think them necessary, and all physicians who are not members of this Association do likewise—verily a distinction without a difference. They only claim to be a little more cautious now than formerly in prescribing alcoholics.

"There is no conclusive evidence that alcohol has any more power in rectifying morbid processes and removing disease than it has in promoting the vital processes in a state of health." The reply to this assertion can be made in a very few words—*i. e.*, all of the whole long list of eminent text-writers quoted above disagree with Dr. Logan on this point. The use of alcoholics in disease rests upon exactly the same fundamental principles as quinine, morphine, arsenic, strychnia, etc.

Quoting Dr. N. S. Davis, the essay says: "That a part at least (and from the testimony of other observers, I would say the whole) of the amount taken is finally eliminated or thrown out of the system with the excretions without having undergone any appreciable chemical change. These facts are as well established as any in the domain of physiology, or in the whole field of natural science, and they point with all the clearness and force of a mathematical demonstration to the conclusion that alcohol is in no sense food, neither furnishing material for the tissues nor fuel for combustion, nor yet generating nervous or muscular power."

Gentlemen, Dr. Davis, in a pamphlet published in 1871 by the National Temperance Association of America, says: "Alcohol undergoes no chemical change in the system, but is eliminated through the excretory organs, more especially the lungs and kidneys, generally within a few hours after it is taken. This position was fully established by the well-devised and carefully executed experiments of Lallemand, Perrin and Duroy." Dr. Davis has evidently changed his opinion on this subject, for in a pamphlet issued in 1876 by the same temperance association he said, "That a part, at least, of the amount taken is finally eliminated or thrown out of the system with the excretions without having undergone any appreciable chemical change." I will show you as this argument proceeds why he changed his opinion. Gentlemen, it was proper a quarter of a century ago for Dr. Davis to have quoted the experiments and opinions of Lallemand, Perrin and Duroy on the elimination of alcohol from the system, for they were at that time uncontroverted. You are aware that these experiments were announced in 1860, and were for a brief period accepted as conclusive by many scientific men and the public generally. The experiments of these eminent Frenchmen were merely qualitative, and were planned and executed in an ungainly manner, and were marked by an almost utter absence of all reasonable precautions against error. Imperfect as they were, they at the time satisfied the professional mind and gave wonderful impetus to the temperance crusade. But the errors in the researches of Lallemand, Perrin and Duroy were soon detected by the scientific minds of Anstie, Dupre, Thudicum, Schulinus, etc., and called forth from them amazing energy, definiteness and accuracy of experiments and honorable regard for scientific truth and honesty in long years of skillful, accurate researches upon the question in all its bearings. The result of the labors of Anstie, Dupre, Thudicum and Schulinus was the absolute demonstration that the researches of Lallemand, Perrin and Duroy were incomplete, inexact, unscientific and therefore unworthy of acceptance by the scientific mind, and we can say of the facts pointed out by these eminent scientists—Anstie, Dupre, Thudicum and Schulinus—in the forcible words of Dr. N. S. Davis when endorsing the views of Lallemand, Perrin and Duroy: "These facts are as well established as any in the domain of physiology or in the whole field of natural science." I state here and now, and challenge its refutation, that the researches of Anstie, Dupre, Thudicum and Schulinus have for a long number of years been accepted by the medical profession as fully and finally conclusive on this subject. These observers showed incontestably that when moderate doses of alcohol are taken into the system it is disposed of in the body, and that only the merest fraction of it is ever eliminated as alcohol. In the medical profession the views of Lallemand, Perrin and Duroy have long since become obsolete, and have been absolutely supplanted by those of Anstie, Dupre and Thudicum. Dr. B. W. Richardson, the celebrated temperance lecturer, the recognized leader of the temperance cause in Great Britain, says in one of his "Ten Lectures on Alcohol," published in 1885 by the National Temperance Society and Publication House of America:

"We are driven by the evidence now before us to the certain conclusion that in the animal body alcohol is decomposed—that is to say, a certain portion of it (and if a certain portion, why not the whole?) is transmutable into new compounds. The inference that might be drawn is fair enough that the alcohol is lost by being burned in the body. It is lost in the body, and out of the body it will burn. If it will burn in the organism it will supply force, for it enters as the bearer of so much potential energy. . . . I believe there is a certain determinable degree of saturation of the blood with alcohol, within which degree all the alcohol is disposed of by its decomposition. Beyond that degree the oxidation is arrested, and then there is an accumulation of alcohol, with voidance of it, in the unchanged state in the secretions." Does anybody accuse Dr. Richardson of being an anti-prohibitionist? Certainly not, for he is the honored and chosen spokesman of the temperance crusade in Great Britain.

Is it not amazing that in this day and generation, when the views of Lallemand, Perrin and Duroy have long since been repudiated by the medical profession and become obsolete—fallen into the deadly sleep of "innocuous dissuetude"—and renounced with disappointment and sorrow by the brightest and best medical minds enlisted in the temperance crusade, those of us who contend for the therapeutic value of alcoholic liquors are asked to accept the fossilized opinions of Lallemand, Perrin and Duroy? The mere statement of the fact demonstrates the painful absurdity of the position of the defenders of the total elimination theory. Is it not respectful and proper to ask of those who mould the temperance sentiment of the people that the medical aspects of the question should be intelligently and fairly presented?

Suppose we present a somewhat brief review of the stages of medical opinion on the elimination of ingested alcohol from the animal body. To fully present this subject in all its bearings it would be proper to incorporate in this paper the contribution of Dr. A. Dupre, entitled "The Physiological Action of Alcohol," *London Practitioner*, vol. ix., 1872, pages 28–34, and that of Dr. Anstie, "Final Experiments on the Elimination of Alcohol from the Body," *London Practitioner*, 1874, p. 15–28. The length of these contributions makes it impossible to present them on the present occasion. I propose to convince the judgment of any honest, intelligent man on this point, and for this purpose shall quote temperance literature. I give you below the statement of Dr. B. W. Richardson, of London, the recognized leader of the temperance cause in Great Britain, and by all odds the most learned medical scientist ever enlisted under the temperance banner. Dr. Richardson says, on the "Disposal of Alcohol in the Organism:"

"The earlier physiologists of this century came naturally enough to the conclusion that the alcohol taken into the body is consumed there with the evolution of heat. A certain development of heat in the superfices of the body, and a certain sensation of glow which follows upon the imbibition of spirit, lent countenance to this suspicion. But in course of time, independently of any knowledge of the effect produced by alcohol in the minute circulation of the blood, it began to be doubted whether alcohol was disposed of in the organism by its combustion. Some observers had noticed, in conducting the examination of the body after death from excess of alcohol, that the odor of the substance was present in the tissues, especially in the nervous tissue, and it was doubted whether the alcohol might not under some circumstances remain in the organism without undergoing any change at all. In 1860 two eminent Frenchmen, Lallemand and Perrin, assisted by Duroy, published a prize essay on alcohol, in which this view was maintained, or, as the authors would probably say, was originated, for in truth they were the first to state the view on direct scientific evidence. From the result of many experiments, they came to the conclusion that alcohol taken into the living body accumulates in the tissues, especially in the liver and in the brain, and that it is eliminated by the fluid secretions, notably the renal secretion, as alcohol. They sought in the different tissues for evidence of the secondary products of the oxidation of alcohol, for aldehyde, acetal, acetic acid, and they found none of these products, except some acetic acid in the stomach, which acid they concluded was formed from the alcohol received directly into the stomach, and from the action exerted upon it there by the gastric juice. The experiments carried on by these inquirers were numerous and careful, and the results they arrived at were so definitely stated that their labors were for a season accepted as conclusive by many men of science and by the majority of the public. It was ascertained by other experimentalists that alcohol is eliminated by the system in the direct way, as alcohol, and the question of elimination rested as if it had been solved."

"The interval of credence in these assertions was not very prolonged. An English physician soon commenced to cross a lance with his learned French peers, and to point out certain distinct errors in their results. I have no doubt many of you know, before I mention his name, that he to whom I refer was the physician who last year lost his life from the performance of his professional duties—the late Dr. Anstie. Respecting this observer, whose friendship I owned for many years, it is meet for me to pay this public tribute of respect, that no man I ever knew combined with vigor of mind more incomparable industry and courage, or a more honorable regard for scientific truth and honesty. The subject we are now considering has lost no investigator more ably learned for the work that still remains to be done."

"From Dr. Anstie came the earliest expressions of doubt relative to this hypothesis of what is called the direct elimination of alcohol by the secretions, and from him have come the latest objections. His arguments have been sustained abroad by Schulinus, and in this country by Drs. Thudicum and Dupre, whose work on wine will, even in another century, be more highly prized, if that be possible, than it is now. The sum and substance of the labors of these observers is stated in a few words. They prove that while it is true that, under certain circumstances, alcohol taken into the body will pass off in the secretions unchanged, the quantity so eliminated is the merest fraction of what has been injected, and that there must be some other means by which the spirit is disposed of in the organism. In a lecture I delivered on this subject in the year 1869, I ventured to suggest, in commenting upon a series of Dr. Thudicum's remarkable researches, that perhaps one element of research was wanting to prove conclusively the fallacy of the direct elimination hypothesis. I thought that sufficient time had not been allowed between the administration of the spirit and the final determination made for it in the excreted fluids. It was not, I argued, shown how much spirit the tissues would hold unchanged. The objection was sound, but it has been removed by more recent experiment."

"In the last research conducted by Dr. Anstie, in which he was assisted by Dr. Dupre, the results of the experiments were unmistakable in their bearing on the points now under our consideration. The history of these labors is recorded in full in the last paper written by Dr. Anstie, and published in the *London Practitioner* for July, 1874."

"The test that had been commonly employed for determining the presence of alcohol in the fluid suspected of containing it was the color test. A solution is made consisting of bichromate of potassa, with diluted sulphuric acid. When to this solution alcohol is added, there is a change of color from the brownish red to green, owing to the reduction of the chromic acid to the green oxide of the base chromium. By marking the difference of color produced, a scale can be adopted which will show the extent of the reduction, and thereby the amount of spirit that has caused the change. This process was improved by Dr. Dupre. He distilled the fluid in which alcohol was believed to be present, and then, after treating the distillate with the bichromate and sulphuric acid solution, he tested with a standard solution of soda for the amount of acetic acid which would be produced by the oxidation of alcohol were that fluid present."

"This modification of test was and is a very considerable advance, since it enabled the observers to extend their determinations with greater accuracy of detail. In the research they conducted with it, two facts of singular interest were elicited. The first fact was discovered by Dr. Dupre. It is that from the secretions of persons who do not drink alcohol at all a fluid can be distilled which affects the chromic acid test as if alcohol were actually present in the secreted fluids, and that this hitherto unsuspected product is oxidized into an acid so like acetic acid it cannot be distinguished from it, and is apparently identical with it. To be plain, Dr. Dupre's discovery suggests that no man can be, in a strict scientific sense, a non-alcoholic, inasmuch as, 'will he nil he,' he brews in his own economy a "wee drop." It is an innocent brew, certainly, but it is brewed, and the most ardent abstainer must excuse it. 'Argal, he that is not guilty of his own death shorteneth not his own life.' The fault, if it be one, rests with nature, who, according to our poor estimates, is no more faultless than the rest of her sex."

"The second fact, which came chiefly from the labors of Dr. Anstie, is that from animals under alcohol, not one of the secretions, not all the secretions combined, yield any more than a fractional amount of the alcohol that has been administered. The experiments were by necessity made on the inferior animals, but they supplied none the less conclusively the fact stated. It was proved that an animal, a terrier dog weighing ten pounds, could take with comparative impunity nearly 2,000 grains of absolute alcohol in ten days, and that on the last day of this regimen he only eliminated by all the channels of elimination 1.13 grains of alcohol. This fact was of itself sufficiently remarkable, but another still more important remains to be told. In completion of his research, when an animal had been treated with alcohol as above described, Anstie killed it, instantly and painlessly, two hours after it had received the last quantity (95 grains) of proof spirits. Then the whole body, including every fragment of tissue with all the fluid and solid contents, was subjected to analysis, with the result of discovering only 23.66 grains of spirit."

"We are driven by the evidence now before us to the certain conclusion that in the animal body alcohol is decomposed—that is to say, a certain portion of it (and if a certain portion, why not the whole?) is transmutable into new compounds. The inference that might be drawn is fair enough that the alcohol is lost by being burned in the body. It is lost in the body, and out of the body it will burn. If it will burn in the organism it will supply force, for it enters as the bearer of so much potential energy."

But suppose you accept as facts the views of Lallemand, Perrin and Duroy, that alcohol is eliminated unchanged from the human body, does such acceptance deny alcoholics a very high place in therapeutics? Certainly not. Do we not know that 96 per cent. of the quinine taken into the system is eliminated by the kidneys within twelve hours after its ingestion, yet who denies that quinine is one of our best known and most reliable therapeutic agents. Many of our best-known and most valuable medicines are eliminated from the body unchanged in greater or less per cent., and we have yet to hear it seriously argued that because of this fact they are to be branded as unreliable in the treatment of disease. The only possible bearing which the elimination of alcohol from the human system can have, if we admit the elimination theory to the fullest extent, is to deny it a place as a food. It is not the purpose of this paper to discuss the food-value of alcoholics, although nearly all the best authorities upon foods assign them such a place. The claim made for the class-alcoholics is that it is a most valuable, indispensable stimulant, which sustains life, as no other medicine can, in adynamic conditions of many of the most frightfully fatal diseases known to man. This claim is made by almost all of the best text-writers in medicine, whose books have ever within the last twenty years received the endorsement of the medical profession in America or in Europe. Some of these text-books are so recently issued that the ink with which they are printed is scarcely dry upon the leaves, and yet temperance crusaders have the brazen impudence to tell those of us who coincide with these learned text-writers, "You do not know the recent literature of your own profession, for the views which you contend for are repudiated by all modern authorities in medicine."

Quoting Dr. J. R. Nichols, the paper under review says: "The distinguished chemist of New England (Dr. J. R. Nichols) says: That if the natural vinous fermentative process should cease and the art of distillation become a lost art, not a life would be sacrificed in consequence, not a case of disease would be retarded in the cure, not a pain would be aggravated, and not one of the art processes suffer detriment."

I have already answered all the points made in the above quotation, except that in relation to the relief of pain. In reply to this point I wish to say, with all the emphasis which I can possibly command, "If the natural vinous fermentative processes should cease and the art of distillation become a lost art," that surgery, resplendent as it is with its marvelous achievements in prolongation and actual savings of human life, would then become a "lost art," for it cannot, and will not, be denied that alcohol is essential to the production of every one of the general anæsthetic agents in our materia medica, except nitrous oxide or laughing gas. If there is anything known in relation to nitrous oxide, it is that in consequence of the intense venosity of the blood which occurs during its use it is unsuitable for continued administration, and therefore inadmissible in a case of lengthy surgical operation.

Furthermore, gentlemen, with the banishment of alcohol from the laboratory our materia medica will be called upon to give up that grand remedy, hydrate of chloral, which has saved thousands upon thousands of lives since its introduction into our medical armamentarium. Other similar facts might be presented, but these are sufficient to demonstrate the utter absurdity of attempting to banish alcohol from the laboratory.

(*To be Continued.*)

